# c-Jun in neurodegeneration: A key transcriptional regulator with therapeutic implications

**DOI:** 10.1016/j.omtn.2026.102874

**Published:** 2026-02-26

**Authors:** Faiz Ali Khan, Hizbullah Khan, Usman Ayub Awan, Mammat Nurahmat, Mohammadtursun Nabijan, Muhammadjan Abduwaki, Jingcheng Dong

**Affiliations:** 1 Institutes of Integrative Medicine, Fudan University, Shanghai 200032, China; 2 Department of Integrative Medicine, Huashan Hospital, Fudan University, Shanghai 200040, China; 3 Guangdong Provincial Key Laboratory of Medical Immunology and Molecular Diagnostics, School of Medical Technology, Guangdong Medical University, Dongguan 523808, China; 4 Department of Medical Laboratory Technology, The University of Haripur, Haripur 22620, Khyber Pakhtunkhwa, Pakistan; 5 Xinjiang Key Laboratory of Hetian Characteristic Chinese Traditional Medicine Research, Xinjiang Hetian College, Hetian 848000, China

**Keywords:** MT: Oligonucleotides: Therapies and Applications, c-Jun, AP-1, neurogenesis, neurodegenerative diseases

## Abstract

c-Jun, a core component of the activating protein-1 (AP-1) transcription factor complex, regulates cellular processes including proliferation, differentiation, survival, apoptosis, and oncogenesis. c-Jun functions by dimerizing to bind DNA and modulates the expression of genes such as Bcl-2, cyclin D1, and pro-inflammatory cytokines, enabling context-dependent transcriptional control. Its role in neurodegenerative diseases has gained attention due to its regulation of oxidative stress, inflammation, and apoptosis. In Parkinson’s disease, Alzheimer’s disease, and Huntington’s disease, dysregulated c-Jun expression accelerates dopaminergic neuron loss via oxidative damage, contributes to amyloid-β-induced synaptic toxicity, and mediates neuronal apoptosis and inflammation, respectively. Despite its degenerative role, c-Jun also promotes axonal regeneration and stress adaptation, revealing a dual function that depends on context and stimulus severity. This paradox underscores its ability to promote survival under mild stress and apoptosis under chronic damage. Emerging therapeutic strategies targeting c-Jun—via small-molecule inhibitors (e.g., SP600125), RNA interference, or modulation of upstream c-Jun N-terminal kinase (JNK)—are being explored. However, challenges remain in achieving specificity, as c-Jun’s ubiquitous expression raises concerns about off-target effects. This review highlights recent advances in understanding c-Jun’s complex role in neurodegeneration and its therapeutic potential, emphasizing its value as both a mechanistic regulator and a target for preserving neuronal integrity in neurodegenerative diseases.

## Graphical abstract

**Figure:**
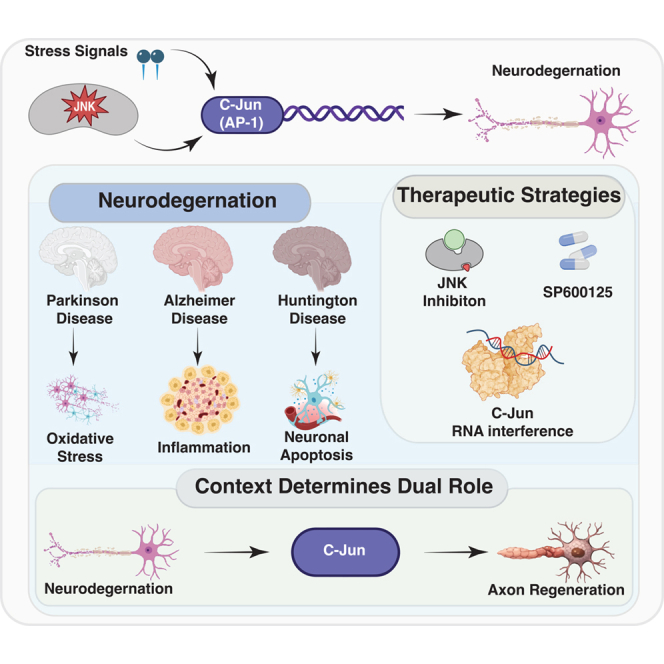

## Teaser

c-Jun emerges as a central transcriptional integrator of neuronal stress in neurodegenerative diseases. This review highlights how diverse pathogenic insults converge on JNK-c-Jun signaling to drive neuronal dysfunction, inflammation, and cell death, while also discussing emerging therapeutic strategies and translational challenges in targeting this pathway.

## Introduction

Activating protein-1 (AP-1) is a dimeric transcription factor (TF) comprising members of the Jun and Fos protein families.^1^ Among the Jun isoforms (c-Jun, JunB, JunD), c-Jun stands out as a central AP-1 component.^2^^,^^3^ Notably, c-Jun is highly expressed in developing, adult, and injured nervous tissue,^4^ consistent with its involvement in controlling cell proliferation, neuronal differentiation, and survival.^4^ Through these gene regulatory effects, c-Jun can influence neural development and orchestrate cellular responses to stress.

Over the past few decades, studies have shown that c-Jun expression and activity are dynamically regulated in various physiological and pathological contexts.^4^ For example, c-Jun levels and phosphorylation increase during embryonic and postnatal neuronal development,^5^^,^^6^ as well as in response to neural stress or injury, such as following transplantation.^7^ In the embryonic brain, c-Jun mRNA is abundantly expressed in progenitor-rich germinal layers around the ventricles,^8^ underscoring its key role in neurogenesis. Collectively, these observations implicate c-Jun as a mediator of diverse neural phenomena, from cell cycle progression and axon growth to programmed cell death.^4^

Despite extensive study, many aspects of c-Jun’s function remain complex and even paradoxical. As a basic leucine zipper (bZIP) protein,^9^ c-Jun functions by dimerizing to bind DNA, and its activity is modulated by post-translational modifications triggered by extracellular signals. Yet, c-Jun can have seemingly opposing effects: in some contexts, it promotes neuron survival and regenerative growth, whereas in others, it contributes to apoptosis and neurodegeneration.^10^ This dual capacity suggests that context-specific co-factors or additional regulatory mechanisms influence how c-Jun drives different outcomes. Furthermore, emerging evidence hints that c-Jun may engage in non-canonical activities beyond its classic transcriptional role, such as interacting with chromatin modifiers or other signaling pathways, which remain to be fully elucidated.

In this review, we examine c-Jun’s structural features and regulatory mechanisms to shed light on its contributions to neurodevelopment, neurogenesis, axonal growth, and neurodegenerative diseases. By synthesizing recent findings, we aim to clarify how c-Jun integrates extracellular cues with gene transcription programs to shape neural circuits and promote repair processes. We also highlight outstanding questions, such as how c-Jun’s activity tips the balance between cell survival and apoptosis, that continue to drive investigation into its diverse functional repertoire.

## c-Jun

c-Jun was originally identified in transformed cells carrying the avian sarcoma virus 17 genome, which produces a 65-kDa gag–jun fusion oncoprotein called v-Jun.^11^ Soon after, the cellular proto-oncogene c-Jun was isolated from human and mouse tissues.^12^ c-Jun shares sequence similarity with the yeast bZIP protein GCN4, and notably, both GCN4 and mammalian AP-1 recognize the same DNA sequence. Using an AP-1 enhancer element as bait in affinity chromatography enabled the co-purification of mammalian c-Jun with c-Fos, leading to the discovery that AP-1 is a heterodimer composed of c-Jun and c-Fos.^13^^,^^14^^,^^15^^,^^16^^,^^17^ As the founding member of the AP-1 family, c-Jun is the most potent transcriptional activator in this group.^18^ Various stimuli, such as the tumor promoter TPA (12-o-tetradecanoylophorbol-13-acetate), growth factors, and cellular stress, markedly enhance c-Jun’s DNA-binding and transcriptional activity.^3^^,^^19^^,^^20^^,^^21^ These extracellular signals initiate signaling cascades that rapidly change c-Jun’s phosphorylation status, independent of new protein synthesis.^18^^,^^22^^,^^23^^,^^24^ Phosphorylation of serine and threonine residues in c-Jun’s N-terminal transactivation domain is critical for its post-translational regulation and typically enhances c-Jun’s transcriptional activity.^25^ Although phosphorylation can trigger AP-1 activation, the phosphorylated c-Jun protein is relatively unstable and transient within the cell,^26^ suggesting that phosphorylation alone is insufficient to sustain prolonged AP-1 activity.^27^ Nonetheless, such modifications alter c-Jun’s affinity for specific gene promoters, thereby influencing gene transcription.

An important aspect of c-Jun regulation is an auto-regulatory feedforward loop: AP-1 activation drives *JUN* gene transcription, and the resulting c-Jun protein, in turn, amplifies AP-1 activity by binding to its own promoter.^3^ This positive feedback mechanism prolongs AP-1 activity and strengthens its transcriptional impact.^16^ Through this loop, c-Jun effectively converts transient upstream signals into sustained AP-1 activation, enabling long-term changes in gene expression.^17^ Consistent with this, persistent AP-1 activation correlates with stably elevated c-Jun levels, reinforcing c-Jun’s central role in the network of AP-1 regulation.

As AP-1 research has expanded, it has become clear that its regulatory mechanisms are highly intricate. AP-1 dimers bind selectively to enhancer elements in certain gene promoters, such as the human metallothionein IIA promoter or the SV40 enhancer. Each AP-1 component exhibits distinct dimerization capabilities: Jun proteins can form either homodimers or heterodimers with Fos or certain activating TF (ATF) family members; ATF proteins form stable homodimers; and Fos proteins cannot homodimerize at all. Despite sharing a common consensus DNA-binding sequence (5′-TGA(C/G)TCA-3′), different AP-1 dimers vary in their DNA-binding affinity and subtle sequence preferences. Moreover, AP-1 expression and activity vary significantly across cell types and tissue contexts.^26^ The cellular context influences AP-1 composition and function, as myriad growth factors, cytokines, hormones, and environmental stressors regulate AP-1 at multiple levels, including *jun*/*fos* gene transcription, mRNA stability, post-translational modifications, and protein degradation. These regulatory mechanisms alter the intracellular abundance or activity of AP-1 components and thereby modulate AP-1’s functional output.^28^ Consequently, AP-1 operates as a complex mixture of dimers with diverse DNA-binding specificities and transcriptional activities. It controls the expression of a vast array of genes involved in cell proliferation, survival, differentiation, and apoptosis, and it plays crucial roles in numerous biological and pathological processes, ranging from epidermal and neural development to immune responses and tumorigenesis. Understanding the AP-1/c-Jun regulatory network and its impact on cellular functions remains a major focus of molecular biology research.

## c-Jun-related signaling pathway

Extracellular signals are transmitted through the cytoplasm via protein kinase cascades that ultimately lead to TF activation or modification.^29^ In the case of c-Jun, several kinases can modify its activity. Glycogen synthase kinase-3 (GSK-3) and casein kinase II (CKII) phosphorylate c-Jun on its C-terminal region, keeping c-Jun in a non-DNA-bound state.^22^ By contrast, the mitogen-activated protein kinase (MAPK) ERK can indirectly enhance c-Jun’s activity: ERK activates p70 S6 kinase, which phosphorylates c-Jun on serine 21—an event that paradoxically inactivates c-Jun’s DNA-binding ability—but this leads to subsequent C-terminal dephosphorylation of c-Jun (by inhibiting GSK-3), thereby ultimately increasing c-Jun’s DNA-binding activity.^30^ Mutational studies in fission yeast established a functional link between an ERK-related pathway and Jun, showing that their mammalian homologs control yeast cell elongation immediately after cell division, suggesting an evolutionarily conserved connection between these signaling molecules.^31^

Among the major signaling pathways that regulate c-Jun, the c-Jun N-terminal kinases (JNKs) are particularly prominent. JNKs, ERKs, and p38 MAPKs constitute three main groups of MAP kinases, all activated by dual phosphorylation of a conserved Thr-X-Tyr motif by their respective MAPK kinases (MKKs) and upstream MAPK kinase kinases (MAPKKKs). Each MAPKKK responds to specific stimuli, thus funneling particular extracellular signals into the MAPK cascades that converge on TFs like c-Jun. The JNK pathway, in particular, is named for its ability to phosphorylate c-Jun on its N-terminus, thereby potently increasing c-Jun’s transcriptional activity. JNK signaling plays a central role in cellular responses to a variety of stress signals and growth factors, while also contributing to basal processes required for tissue homeostasis. It can regulate cell proliferation, differentiation, migration, and apoptosis in a highly context- and cell-type-specific manner.^32^^,^^33^^,^^34^ When dysregulated, JNK signaling—and consequently c-Jun activity—can contribute to pathological conditions such as chronic inflammation, tumorigenesis, and neurodegeneration.^34^ As a stress-activated protein kinase (SAPK), JNK is activated by numerous stressors, such as heat shock, UV irradiation, pro-inflammatory cytokines, and oxidative stress. Upon activation, JNK directly phosphorylates components of the AP-1 TF complex, particularly c-Jun, JunD, ATF2, and c-Fos, thereby modulating the expression of various AP-1 target genes.^35^^,^^36^^,^^37^^,^^38^

**Figure 1:**
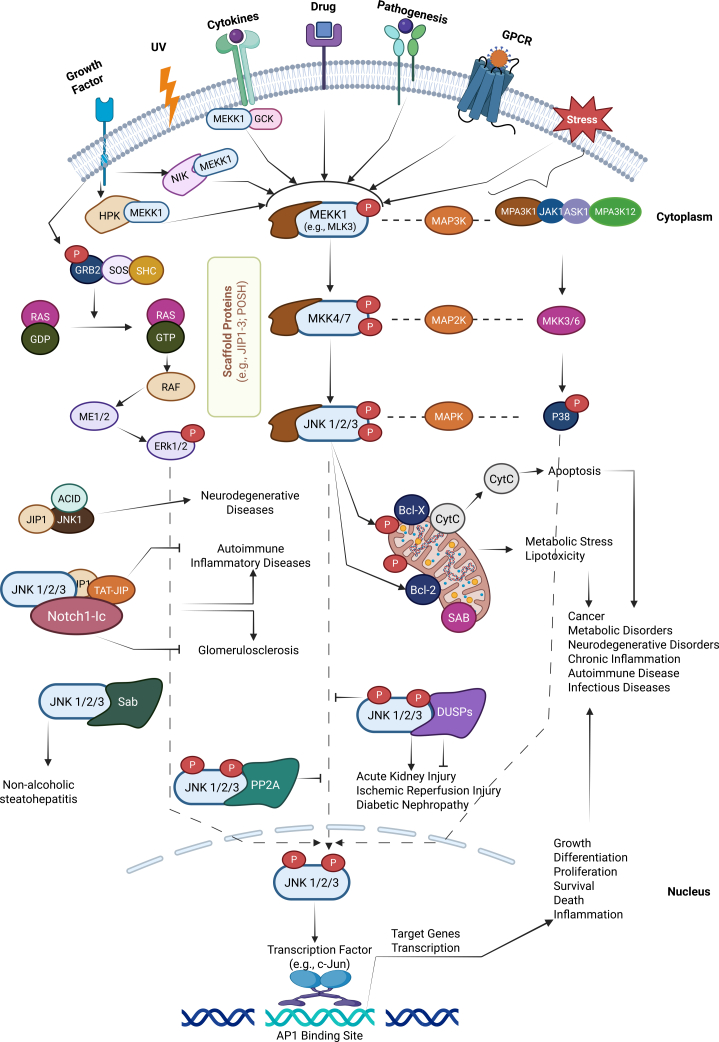
The general activation mechanism of the JNK signaling pathway

In neurons, JNK-mediated regulation of c-Jun is especially significant. The mammalian brain exhibits high JNK activity, with the JNK3 isoform being predominantly expressed in the central nervous system (CNS), whereas JNK1 and JNK2 are ubiquitously expressed in many tissues.^39^^,^^40^ JNK3 is strongly implicated in neuronal stress responses: aberrant JNK3 activity has been linked to synaptic dysfunction and is thought to act as an early driver of neurodegenerative and neurodevelopmental disorders via excessive c-Jun activation.^41^ Thus, in the nervous system context, JNK3-driven c-Jun phosphorylation is a key event that can tip the balance toward neuronal death or other alterations in cell fate. This has made the JNK–c-Jun axis a potential therapeutic target in neurodegenerative disease research. Indeed, blocking JNK activation or c-Jun function has shown neuroprotective effects in various experimental models, underscoring the importance of this pathway in neuronal pathology (Figure 1).

## c-Jun-related regulation

As one of the most extensively studied AP-1 proteins, c-Jun plays crucial roles in many cellular processes, including cell proliferation, apoptosis, survival, and tumorigenesis. Early studies on c-Jun’s structure and function identified it as a bZIP DNA-binding protein.^28^ Subsequent work demonstrated that extracellular stimuli can trigger post-translational modifications of c-Jun, thereby altering its transcriptional activity and the expression of its target genes.

Recent investigations have revealed a complex regulatory framework in which c-Jun orchestrates crosstalk and amplification across multiple signaling pathways essential for tissue growth and disease progression. One prominent mechanism is the autocrine amplification loop described earlier: stimulus-induced AP-1 activation upregulates *c-Jun* gene expression, and the resulting c-Jun protein further enhances AP-1 activity by binding to AP-1 sites in its own promoter. This self-reinforcing loop greatly extends c-Jun’s transcriptional impact and solidifies its role in gene regulation. Additionally, studies in knockout mouse models have shown that c-Jun integrates signals from multiple developmental pathways, including EGFR-ERK, EGFR-RhoA-ROCK, and activin B-MAP3K1-JNK cascades, to mediate certain morphogenetic processes such as embryonic eyelid closure.^3^ In these contexts, c-Jun acts as a point of convergence for disparate signaling inputs, translating them into the gene expression programs needed for proper tissue development.

At the molecular level, c-Jun is subject to intricate control. Phosphorylation at key serine and threonine residues modulates c-Jun’s stability and transcriptional function.^42^^,^^43^ The JNK pathway is a major upstream regulator that phosphorylates c-Jun, thereby influencing AP-1 activity and downstream gene expression. Conversely, other modifications, such as C-terminal phosphorylation by GSK-3 or CKII, can dampen c-Jun’s DNA binding. c-Jun also interacts with various co-regulatory proteins, including the CBP/p300 acetyltransferase coactivators, which can acetylate c-Jun to enhance c-Jun-driven transcription.^44^ Through such post-translational modifications and protein-protein interactions, c-Jun serves as a hub that integrates diverse signaling cascades and modulates cell-fate decisions.

Even after decades of research, c-Jun remains a central component of a vast molecular network with intricate functional properties. New aspects of c-Jun regulation and function continue to be uncovered, highlighting its versatility. For instance, c-Jun’s activity can be influenced by other transcriptional networks: the *JUN* promoter contains binding sites for factors such as NF-κB, SP1, and C/EBP, in addition to AP-1, indicating that multiple signaling pathways can feed into *JUN* expression. Such insights underscore how c-Jun links various cellular signals to long-term biological outcomes. Elucidating these mechanisms may offer novel therapeutic targets for diseases linked to aberrant c-Jun activity, including certain cancers, neurodegenerative disorders, and inflammatory conditions (Figure 1).^45^^,^^46^

## c-Jun and neurogenesis

Beyond its general roles, c-Jun has specific functions in the nervous system, particularly in neural development and neurogenesis. During embryonic development, c-Jun is highly expressed in proliferative neural precursor populations, supporting its established role in developmental neurogenesis. In contrast, c-Jun expression in the adult brain is more restricted and context-dependent, reflecting fundamental differences between embryonic neurodevelopment and adult neural homeostasis. These observations suggest that c-Jun is crucial for neural progenitor cell (NPC) proliferation during development and may have distinct functions in adult neurogenesis.^8^

Postnatally, c-Jun expression persists in restricted neurogenic regions, but its spatial distribution and functional relevance differ markedly from those observed during embryogenesis.^47^^,^^48^ These findings support a developmental role for c-Jun in proliferative neural programs rather than widespread regulation of cell division in the adult brain.

In the adult subventricular zone (SVZ)-rostral migratory stream-olfactory bulb (SVZ-RMS-OB) system, c-Jun appears to have complex and context-dependent roles. It may help regulate the migration or differentiation of adult NPCs, as adult SVZ stem cells exhibit region-specific characteristics and differentiation potentials.^49^ c-Jun might also function differently across subpopulations of adult NPCs. Although c-Jun has been linked to apoptotic signaling under stress conditions, available evidence suggests that its role in adult neurogenic niches is not primarily associated with widespread cell death.^50^^,^^51^ Rather, c-Jun may influence selective aspects of NPC behavior, such as migration or differentiation, in a highly context-dependent manner.

In summary, c-Jun exerts distinct functions across developmental stages, with a well-established role in embryonic neurogenesis and more limited, context-specific activity in adult neurogenic regions.^52^ Importantly, in the context of neurodegenerative diseases, c-Jun activity is more commonly associated with stress-responsive signaling and neuronal injury rather than active neurogenesis or cell replacement.

## c-Jun and neuronal death

One hallmark of neuronal cell stress and impending death is the activation of the JNK pathway and the rapid upregulation of its downstream target, c-Jun. In various models of neuronal injury, JNK-mediated phosphorylation of c-Jun on its N-terminal transactivation domain, primarily at Ser63 and Ser73, is observed, markedly enhancing c-Jun’s transcriptional activity.^53^^,^^54^ While JNK pathway activation is necessary for neuronal apoptosis in many cases, it is noteworthy that healthy neurons can exhibit high JNK activity without undergoing apoptosis.^55^^,^^56^^,^^57^ This suggests that JNK signaling has functions beyond inducing apoptosis and that additional checkpoints or cofactors, such as c-Jun phosphorylation, determine whether a neuron lives or dies. Therefore, identifying the proteins and pathways controlled by JNK-dependent c-Jun phosphorylation is important for understanding the diverse outcomes of JNK signaling in neurons.

Research has demonstrated that c-Jun itself is a pivotal executor of the apoptotic program in certain types of neurons. For example, in sympathetic neurons deprived of nerve growth factor, there is a rapid induction of c-Jun expression and N-terminal phosphorylation. Blocking c-Jun function by microinjecting neutralizing antibodies into sympathetic neurons significantly delays NGF-withdrawal-induced apoptosis.^58^ Similarly, overexpressing a dominant-negative c-Jun or genetically deleting c-Jun in cultured neonatal sympathetic neurons provides protection and delays cell death, underscoring c-Jun’s role in promoting apoptosis.^53^^,^^59^ However, it remains under investigation whether c-Jun activation by itself is sufficient to trigger neuronal apoptosis or whether it mainly acts in concert with other pro-apoptotic signals.

Multiple *in vitro* models have been used to dissect c-Jun’s role in neuronal death.^49^^,^^53^^,^^59^^,^^60^ Interestingly, in some non-neuronal cell types, c-Jun has been reported to have protective effects against cell damage,^61^ illustrating that c-Jun’s function can be context-dependent. In neurons, however, phosphorylated and activated c-Jun tends to drive the expression of genes that facilitate apoptosis. For instance, mutating the N-terminal Ser63/Ser73 phosphorylation sites of c-Jun substantially inhibits apoptosis in sympathetic neurons, likely because it impairs the induction of *c-Jun’s* pro-apoptotic target genes, including *JUN* itself.^17^ Under normal conditions, neurons express only low levels of c-Jun and show little or no detectable N-terminal phosphorylation of c-Jun, even though upstream JNK activity might be constitutively high. In various models of neuronal stress or injury, however, specific JNK isoforms, such as JNK3 in the brain, become strongly activated and can translocate to the nucleus or other cellular compartments, where they phosphorylate c-Jun at Ser63 and Ser73. This modification increases c-Jun’s stability and transcriptional activity, leading to a sustained, self-amplifying increase in *JUN* gene expression and further c-Jun phosphorylation.^62^ In essence, once c-Jun is activated in a neuron, it can initiate a feedforward loop that reinforces the apoptotic program.

The Bcl-2 family of apoptosis-regulating proteins also governs neuronal survival and death. The pro-apoptotic Bcl-2 family member Bax is indispensable for neuron death in both sympathetic and cerebellar granule neurons.^63^^,^^64^ Upstream of Bax, “BH3-only” proteins serve as sentinels that respond to stress signals by activating Bax. Two *BH3*-only proteins, *Bim* and *DP5/Hrk*, are observed to increase in expression in sympathetic and granule neurons undergoing apoptosis following trophic factor deprivation.^65^ There is evidence that c-Jun contributes to *Bim* upregulation in these models. Indeed, neurons with mutations that prevent c-Jun N-terminal phosphorylation show blunted induction of *Bim* and a delayed onset of apoptosis. However, even in those neurons, cell death is not completely averted—it is delayed but eventually proceeds, indicating that JNK/c-Jun signaling is a critical facilitator of apoptosis rather than the sole cause.

Moreover, DLK (dual leucine zipper kinase) was identified in 2009 as a key regulator of axonal regeneration after laser axotomy in adult neurons. DLK activates JNK, which phosphorylates c-Jun to mediate axonal degeneration and neuronal apoptosis,^66^^,^^67^ underscoring the DLK-JNK-c-Jun axis in balancing regeneration and degeneration during development.^68^

In humans, DLK is activated as a stress response to various injury signals and plays a key role in axonal injury. Through the scaffold protein JIP (JNK-interacting protein), DLK signals via MKK4/7 to activate the JNK pathway, leading to phosphorylation of c-Jun and other TFs, including STAT3, ATF3, KLF6, and TF2, as well as induction of SPRR1A, collectively promoting axon regeneration.^69^ However, persistent DLK-JNK signaling can also trigger axonal degeneration and neuronal apoptosis in mammals.^70^ The MKK4/7-JNK-c-Jun cascade, mediated through the JIP scaffold, is thus central to both regeneration and degeneration, depending on cellular context.^66^^,^^71^ Additionally, JNK-mediated phosphorylation of c-Jun and other TFs, such as TF2, contributes to axonal breakdown under pathological conditions.^72^ In summary, JNK-mediated phosphorylation of c-Jun’s N-terminus plays a vital role in the apoptotic program of neurons by driving the expression of pro-death genes, but it acts in concert with other pathways to fully execute neuronal apoptosis.

## c-Jun and neuronal differentiation

Beyond its pro-apoptotic role, c-Jun is deeply involved in neuronal differentiation and maturation. The JNK/c-Jun pathway plays important roles in neurite outgrowth, synapse formation, and other aspects of neural development and maturation.^73^^,^^74^^,^^75^^,^^76^ In particular, active JNK signaling can directly influence neuronal differentiation and synaptic growth by phosphorylating TFs, such as c-Jun, which in turn regulate genes involved in these processes. When c-Jun is phosphorylated by JNK, it becomes a key regulator of neuronal cell fate and viability by driving the expression of genes that promote neurite extension and neuronal maturation.^27^^,^^77^^,^^78^

Notably, in the adult brain and in neurodegenerative disease contexts, new neuron generation from neural stem or progenitor cells is very limited.^79^ Thus, c-Jun’s pro-differentiation functions are chiefly evident in developmental or regenerative models rather than under normal adult conditions. Nevertheless, understanding how c-Jun regulates neuronal differentiation could enable researchers to harness this pathway to promote neuronal replacement or repair in degenerative conditions. Indeed, enhancing the neuronal differentiation of transplanted neural stem cells (NSCs) has been shown to significantly improve functional recovery after brain injury, such as stroke.

Importantly, c-Jun does not act alone in this context; it sits at the intersection of multiple signaling pathways that govern NSC fate. For example, JNK/c-Jun signaling can converge with other developmental pathways, such as Wnt and Notch, which are well-known regulators of neural differentiation.^80^ c-Jun helps integrate these signals and orchestrates gene expression programs that bias NSCs toward neuronal lineages. By modulating JNK activity or c-Jun function, researchers aim to coax NSCs to differentiate more efficiently into neurons. Such strategies could improve the success of stem cell-based therapies in stroke and possibly spinal cord injury.^81^ In summary, c-Jun serves as one of the pivotal TFs linking extracellular cues to the transcriptional program of neuronal differentiation. A deeper understanding of how c-Jun and its partners coordinate this process will be crucial for developing regenerative medicine strategies and improving neural repair mechanisms.

## c-Jun and axonal growth

In the adult CNS, injured neurons have a very limited capacity for axonal regeneration, severely restricting functional recovery after injuries such as spinal cord lesions. One factor contributing to this failure is that mature CNS neurons often do not reactivate the transcriptional programs needed for axon growth.^82^^,^^83^ Targeting TFs is a promising strategy to induce a pro-regenerative state, since a single TF can regulate multiple regeneration-associated genes (RAGs) that promote axon regrowth.^84^^,^^85^ Indeed, forced expression of certain TFs in injured CNS neurons has been shown to induce axon regrowth.^86^ However, the extent of regrowth achieved by these interventions so far has been limited, indicating that new approaches or combinations of factors are needed to more robustly activate the regenerative program. Many genes and pathways that promote peripheral nerve regeneration have been identified; their effectiveness in the CNS context remains largely unexplored or insufficient.

c-Jun has emerged as a key TF in the context of axonal injury responses. In peripheral neurons, such as dorsal root ganglion (DRG) sensory neurons, c-Jun is strongly upregulated after injury and activates numerous RAGs needed for axon regeneration. Given c-Jun’s involvement in diverse cellular processes, it can dimerize with various partner proteins, such as Fos or members of the ATF family, and bind different DNA motifs. Danzi et al.^87^ hypothesized that different c-Jun-containing dimer combinations bind distinct DNA motifs, thereby mediating specific transcriptional programs after axonal injury. Genome-wide analyses of c-Jun binding in injured neurons showed that some changes in motif usage were observed; however, the majority of binding sites still contained the classic AP-1 consensus (TRE or CRE) or in some cases, no clearly recognizable motif. This suggests that a broad increase in chromatin accessibility and overall AP-1 (Jun) activity—rather than new DNA sequence preferences—underlies Jun’s pro-regenerative functions. Additionally, c-Jun dimerization with ATF3 enhances axonal growth in both peripheral and central neurons, indicating that Jun-ATF3 interaction is an important component of the injury-induced gene expression program.^88^

In adult neuron cultures, c-Jun overexpression drives robust neurite outgrowth in certain cell types, such as adult DRG neurons and immature hippocampal neurons, but is far less effective in mature cortical neurons.^89^ Comparative gene expression profiling identified STAT3 as another key pro-regenerative factor that is strongly induced in peripheral neurons after injury and contributes to their regenerative success.^90^ For a long time, technical limitations meant CNS studies could only test one genetic factor at a time, such as overexpressing *Jun* or *Stat3* alone.^90^^,^^91^ Recently, new methods have allowed introducing multiple genes or modulators simultaneously into neurons.^92^^,^^93^^,^^94^ Using such multi-gene approaches, one study tested the overexpression of nine candidate pro-regenerative genes, alone and in pairs, in adult cortical neurons. Strikingly, co-expressing *JUN* with *STAT6* yielded significantly greater neurite outgrowth than expressing any single factor alone, indicating a synergistic pro-regenerative effect of these two TFs.^88^^,^^95^^,^^96^

Consistent with these findings, organotypic cortical slice cultures with c-Jun overexpression, with or without STAT6, also showed enhanced neurite outgrowth. Because c-Jun binds AP-1 sites as a homo- or heterodimer, its overexpression likely activates a broad range of RAGs. Interestingly, neurons with forced c-Jun expression showed improved survival compared to controls and exhibited markedly higher c-Jun mRNA and protein levels.

However, two canonical RAGs, *GAP-43* and integrin α7 (*ITGA7*), remained unchanged at the mRNA expression level despite the enhanced outgrowth. This suggests that c-Jun-driven axonal growth can occur without upregulating those particular RAGs, implying a mechanism distinct from the peripheral nerve regeneration program.^2^^,^^4^^,^^88^ Overall, c-Jun’s ability to stimulate axonal growth underscores its critical role in neuronal regeneration. These insights pave the way for TF-based research strategies to promote axon regeneration and functional recovery in the injured CNS.

## c-Jun and neurodegenerative diseases

Despite the distinct genetic, molecular, and pathological origins of major neurodegenerative diseases, a unifying feature is the convergence of diverse cellular stressors on common stress-activated signaling pathways. Oxidative stress, protein misfolding, mitochondrial dysfunction, and neuroinflammation consistently activate the JNK pathway, leading to phosphorylation and dysregulation of c-Jun. Aberrant c-Jun activation functions as a downstream transcriptional integrator of these insults, promoting shared pathological outcomes across diseases, including neuronal apoptosis, synaptic dysfunction, and amplification of inflammatory signaling. This convergence positions c-Jun not as a disease-specific factor but as a central mediator of neurodegenerative stress responses. Degenerative diseases of the CNS, such as Parkinson’s disease (PD), Alzheimer’s disease (AD), Huntington’s disease (HD), amyotrophic lateral sclerosis (ALS), and multiple sclerosis (MS), involve the degeneration of neurons or supporting glial cells in the brain or spinal cord. Given the limited regenerative capacity of the adult CNS, NSCs have been explored as a potential therapy for neurodegenerative diseases, stroke, and spinal cord injury due to their ability to self-renew and generate new neural cells.^97^^,^^98^^,^^99^ Preclinical studies using NSCs are promising, but significant challenges remain in translating these findings into effective treatments.

In the adult mammalian brain, active neurogenesis is largely restricted to the subventricular zone of the lateral ventricles and the subgranular zone (SGZ) of the hippocampal dentate gyrus. Despite the presence of NSCs in these areas, their capacity to regenerate new functional neurons *in vivo* is limited.^79^ Therefore, a critical strategy in CNS regeneration is to identify factors that stimulate neuronal differentiation of resident or transplanted NSCs.

Various molecules have been investigated for their ability to induce NSC differentiation. For example, retinoic acid accelerates NSC differentiation by upregulating key neurogenic TFs, such as *Neurogenin1* and *NeuroD1*, and signaling molecules involved in neurogenesis.^100^ Growth factors, such as *NGF* and insulin-like growth factor (*IGF*), promote NSC differentiation by activating cell survival and maturation pathways, including PI3K/Akt, MAPK/ERK.^101^^,^^102^ Brain-derived neurotrophic factor and fibroblast growth factors also support neurogenesis by modulating synaptic plasticity and enhancing neuronal survival.^103^^,^^104^

Elevated levels of DLK-JNK pathway components, including phosphorylated JNK (*p*-JNK), p-c-Jun, total JNK, and total c-Jun, have been observed in lumbar spinal cord lysates from patients with sporadic ALS. Additionally, Jiang et al.^105^ reported that *DLK* deletion in sensory neurons or pharmacological inhibition alleviated pain behavior and cystitis pathology in cyclophosphamide (CYP)-induced mice, implicating c-Jun signaling in CYP-induced cystitis pathogenesis.

Future research must address challenges such as optimizing differentiation protocols, improving the survival of newly generated neurons, and integrating transplanted cells into neural circuits. A deeper understanding of NSC differentiation mechanisms is crucial, as is the identification of novel factors that can promote neuronal differentiation. In this context, TFs such as c-Jun, which influence both cell survival and differentiation, could become key targets for CNS regeneration therapies.

## c-Jun in AD

AD is the most prevalent neurodegenerative disorder, marked by progressive memory loss, cognitive impairment, and widespread neuron loss. It affects a significant proportion of the elderly population, with a global prevalence of approximately 40.19%.^106^^,^^107^ In AD, the gradual cognitive decline is associated with activation of stress-related signaling pathways, notably the JNK pathway in the MAPK family. Excessive JNK activity contributes to synaptic loss, oxidative stress, brain atrophy, and neuronal dysfunction. While current therapies can alleviate symptoms, AD remains incurable.

AD pathology is defined by two classic hallmarks: extracellular amyloid-β (Aβ) plaques and intracellular neurofibrillary tangles (NFTs) of hyperphosphorylated tau protein.^108^^,^^109^^,^^110^ Multiple hypotheses have been proposed to explain AD etiology, including the amyloid cascade, tau propagation, cholinergic, mitochondrial dysfunction, and inflammatory hypotheses, with the amyloid cascade hypothesis being the most widely accepted, as it explains many key features of AD.^111^ In this model, amyloid precursor protein (*APP*) is cleaved by specific secretases to generate Aβ peptides, which aggregate into toxic plaques.^112^^,^^113^ Tau protein normally stabilizes microtubules in axons, but in AD it becomes aberrantly phosphorylated and dissociates from microtubules, forming insoluble filaments. This tau pathology destabilizes the cytoskeleton and contributes to neuronal dysfunction and death.^114^^,^^115^^,^^116^ Notably, the burden of Aβ plaques alone does not fully account for the severity of cognitive decline, indicating that multiple factors drive neurodegeneration in AD.

Various extracellular and intracellular stressors in AD, such as Aβ toxicity, tau-induced cytoskeletal disruption, and oxidative damage, activate intrinsic apoptotic pathways.^117^ The Bcl-2 protein family tightly regulates these pathways; it includes pro-apoptotic effectors (*Bax*, *Bak*, *Bok*), pro-apoptotic BH3-only members (*Bad*, *Bid*, *Bim*, *Puma*, etc.), and anti-apoptotic proteins (*Bcl-2*, *Bcl-x_L*, *Bcl-w*, *Mcl-1*, *Bfl-1*).^118^ Stress kinases, including JNK and p38, can shift this balance toward apoptosis, in part through c-Jun-dependent modulation of Bcl-2 family members.^119^ For example, JNK-mediated phosphorylation of Bcl-2 and Mcl-1 impairs their anti-apoptotic function, and JNK activation, likely via c-Jun/AP-1, can suppress *Bcl-2* expression, thereby accelerating neuronal apoptosis.^119^^,^^120^ Pathological tau hyperphosphorylation can further engage the JNK/c-Jun pathway, exacerbating neuronal toxicity.^120^ Consistent with these mechanisms, early AD pathogenesis is marked by active JNK/c-Jun signaling. For instance, phosphorylated JNK—and by extension activated c-Jun—is found in postsynaptic dendritic spines, correlating with synaptic loss.^41^ Elevated levels of phosphorylated JNK3, a kinase that activates c-Jun, also correlate with AD progression in human brains.^121^^,^^122^ Moreover, both *in vivo* and *in vitro* AD models show that dampening JNK/c-Jun activity, through genetic knockdown or pharmacological inhibition, attenuates synaptic loss, reduces Aβ accumulation, and decreases neuronal death.^123^^,^^124^^,^^125^

**Figure 2:**
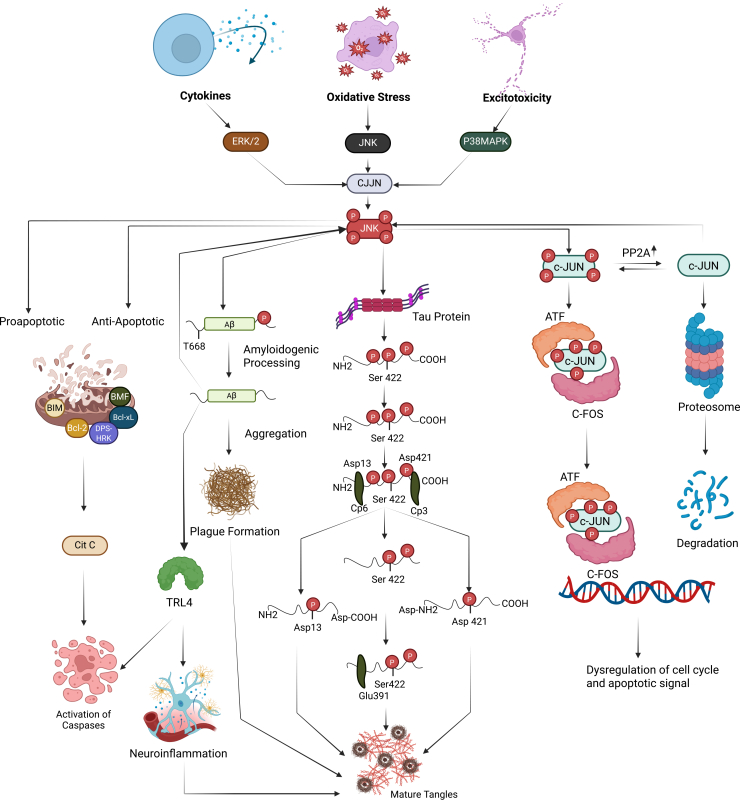
JNK pathway in AD Cellular mechanisms activated by JNK phosphorylation are shown. Activated JNK phosphorylates c-Jun, altering gene expression and promoting tangle maturation. JNK also directly phosphorylates tau, contributing to paired helical filament (PHF) formation and proteolytic processing.

Neuroinflammation has emerged over the past two decades as another key contributor to AD pathogenesis. In AD, chronic inflammation in the brain is largely driven by microglia, the resident immune cells of the CNS.^126^^,^^127^ Microglia play a dual role: on one hand, they can internalize and help clear Aβ deposits via Toll-like receptors (e.g., TLR2- and TLR4-mediated uptake and degradation)^128^; on the other hand, activated microglia secrete pro-inflammatory mediators that can aggravate neurodegeneration. These mediators include interleukins (IL-1β, IL-6), tumor necrosis factor-α (TNF-α), prostaglandin E_2_, nitric oxide (NO), and even aberrant amounts of trophic factors such as brain-derived neurotrophic factor, factors that collectively stimulate the JNK pathway. JNK, in turn, influences the inflammatory response largely through c-Jun/AP-1, which drives the expression of pro-inflammatory genes such as *COX-2*, *NOS2* (*iNOS*), *TNF-α*, *CCL2*, and *VCAM-1*.^129^ Additionally, microglial-derived IL-1β can activate neuronal p38 MAPK signaling, disrupting synaptic and cytoskeletal dynamics.^129^^,^^130^ In essence, Aβ accumulation, tau pathology, and oxidative stress converge on the JNK/c-Jun and p38 pathways, leading to downstream effects such as NF-κB activation, glutamate excitotoxicity, and chronic neuroinflammation (Figure 2).

**Figure 3:**
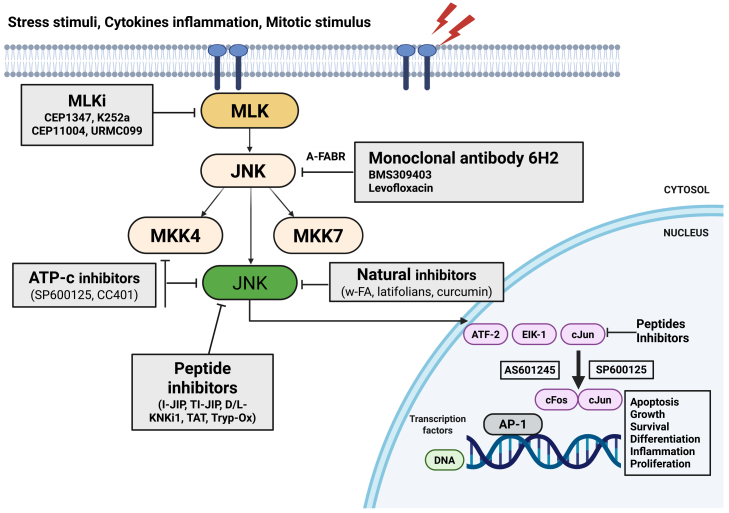
Schematic representation of the mechanism and targets of JNK inhibitors CPPi, cell-permeable peptide inhibitors; ATP-c, direct ATP-competitive inhibitors; MLKi, mixed lineage kinase inhibitors.

Together, these findings establish the JNK/c-Jun signaling axis as a key driver of neurodegeneration in AD, making it a promising target for early diagnostic markers and therapeutic intervention. In experimental models, inhibiting this pathway has yielded neuroprotective effects. For example, the first synthetic JNK inhibitor, SP600125, effectively prevented Aβ-induced neuronal death in AD models.^124^ However, SP600125 is an ATP-competitive inhibitor with limited specificity and known off-target effects on other kinases.^37^ Despite such limitations, studies using various JNK inhibitors have been invaluable in elucidating how JNK/c-Jun contributes to AD pathology. Recently, new JNK inhibitors with improved selectivity and brain penetration have been developed and tested in AD models.^37^^,^^124^ Further research is needed to confirm their safety and specificity, but these next-generation compounds hold promise for dampening c-Jun-mediated neurotoxic processes in AD (Figure 3). Nevertheless, because ATP-competitive JNK inhibitors may affect multiple kinases and JNK isoforms, improved selectivity and brain-penetrant, isoform-biased approaches are likely necessary to reduce off-target effects in chronic neurodegenerative settings.

## c-Jun in HD

HD is a fatal, autosomal-dominant neurodegenerative disorder primarily affecting neurons in the striatum and cortical projection neurons.^131^ This neuronal degeneration leads to severe brain atrophy and a progressive decline in motor, cognitive, and behavioral functions. The disease typically manifests between 35 and 45 years of age, and most patients ultimately succumb to complications of extensive neurodegeneration.^132^^,^^133^

**Figure 4:**
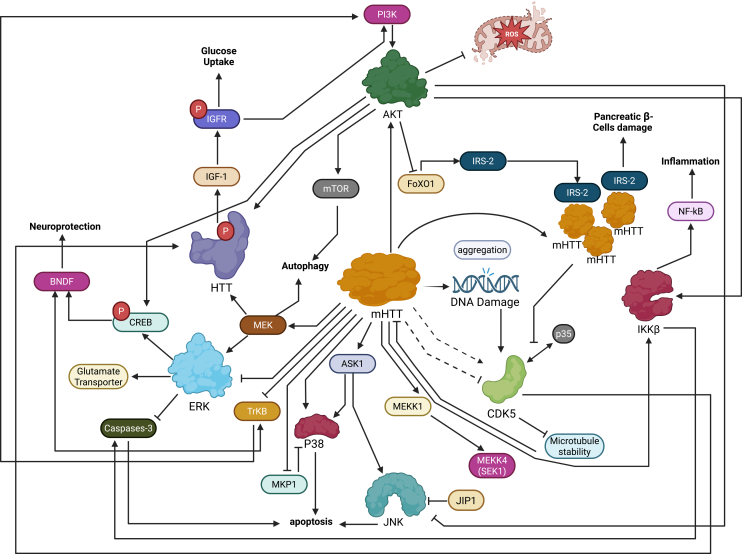
Role of the JNK pathway in HD Molecular crosstalk of mHTT aggregation, DNA damage, and cell death pathways in HD is depicted.

At the molecular level, HD is caused by an expanded CAG trinucleotide repeat in the huntingtin (*HTT*) gene, which produces an abnormally long polyglutamine tract in the HTT protein.^131^ The mutant HTT protein misfolds, aggregates in neurons, and induces cellular toxicity. Notably, mutant polyQ-HTT triggers a cellular stress response that strongly activates the JNK pathway.^134^ JNK, once activated by upstream kinases (such as *ASK1* via *MKK4/7*) under stress conditions, phosphorylates c-Jun on Ser63 and Ser73, greatly amplifying c-Jun’s AP-1 transcriptional activity.^78^^,^^135^^,^^136^^,^^137^^,^^138^ In HD models, JNK3 becomes aberrantly hyperactive, leading to excessive c-Jun activation. Beyond altering gene expression, this hyperactive JNK also phosphorylates cytoskeletal motor proteins, causing microtubule network dissociation and impaired axonal transport in neurons.^139^ Indeed, inhibiting the JNK/c-Jun pathway in HD models has been shown to be neuroprotective. In a rat model, a JNK inhibitor significantly reduced striatal neuron loss and improved motor function by blocking c-Jun activation.^140^ These findings underscore the pathogenic role of aberrant c-Jun activation in HD, highlighting the JNK-c-Jun pathway as an attractive target for therapeutic intervention (Figure 4).

Despite the strong rationale for targeting JNK/c-Jun in HD, the clinical translation of JNK inhibitors has been challenging due to issues of specificity and toxicity. Early broad-spectrum JNK inhibitors lacked isoform selectivity and had off-target effects on other kinases, raising safety concerns. However, advances in structural biology and medicinal chemistry have enabled the development of more selective JNK inhibitors that exploit subtle differences among JNK isoforms (Figure 3). These next-generation inhibitors exhibit higher affinity for specific JNK isoforms, such as *JNK3*, which is predominantly expressed in the brain, providing a more targeted means to diminish pathological c-Jun activity. Ongoing research is evaluating the efficacy and safety of these selective inhibitors in preclinical HD models. If successful, such compounds could lead to novel treatments aimed at slowing HD progression by mitigating c-Jun-mediated neurodegenerative processes. A major remaining limitation is balancing pathway suppression with preservation of physiological stress-response signaling, particularly given the risk of toxicity from sustained inhibition in the CNS.

## c-Jun in PD

PD is one of the most common neurodegenerative disorders, affecting over one million individuals in North America and several million worldwide.^141^^,^^142^ Its primary pathological hallmark is the progressive loss of dopaminergic neurons in the substantia nigra pars compacta, which leads to severe depletion of dopamine in the striatum and gives rise to the characteristic motor symptoms of PD. While the precise pathogenic mechanisms of PD are not fully understood, epidemiological studies have identified various environmental factors, such as chronic pesticide exposure and heavy metal toxicity, that significantly increase the risk of developing PD.^143^^,^^144^^,^^145^

The JNK cascade has emerged as a key contributor to dopaminergic neuron death in PD. Mixed-lineage kinase (*MLK*) family enzymes activate JNK via *MKK4/7*, and activated JNK, in turn, phosphorylates c-Jun and other AP-1 factors, elevating their activity under cellular stress.^146^

In PD models, aberrant JNK/c-Jun activation is a central driver of dopaminergic neuron loss. For example, in the MPTP mouse model, strong JNK activation and c-Jun phosphorylation occur in degenerating nigral neurons and play a pivotal role in their apoptosis.^147^^,^^148^^,^^149^ Similarly, inducing oxidative stress, for example by silencing superoxide dismutase in neurons, hyperactivates JNK/c-Jun and causes greater neuronal damage.^150^ These toxic stressors also activate upstream kinases, such as MKK4, reinforcing the link between oxidative injury and c-Jun-mediated neuronal death.^148^^,^^150^^,^^151^ Consistently, both cell-based and animal studies show that *JNK3*, the brain-enriched JNK isoform, is highly activated in the substantia nigra during PD neurotoxicity.^151^

**Figure 5:**
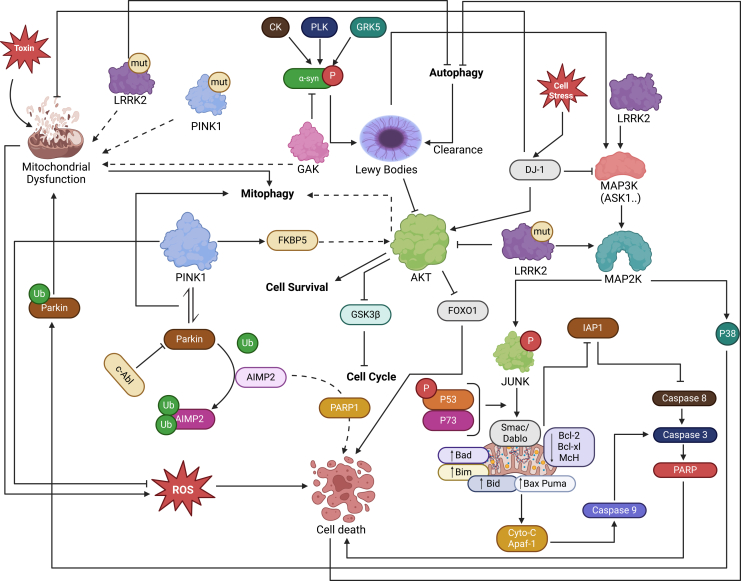
Role of the JNK pathway in PD This figure depicts the signaling networks underlying Parkinson’s disease, with emphasis on the JNK pathway and its contribution to neuronal apoptosis.

Once activated, the JNK/c-Jun pathway promotes dopaminergic neuron death by shifting the balance of pro- and anti-apoptotic signals. c-Jun/AP-1 upregulates pro-apoptotic genes, such as *Bax* and certain *BH3*-only proteins, while JNK simultaneously inhibits anti-apoptotic factors, including *Bcl-2* (through phosphorylation or reduced expression), tipping cells toward apoptosis and even autophagy.^152^ Accordingly, inhibition of the JNK/c-Jun pathway is neuroprotective in PD models: JNK inhibitors prevent dopaminergic neuron loss and ameliorate motor deficits, underlining the therapeutic potential of targeting this stress pathway (Figure 5).^153^^,^^154^ Additionally, dopamine signaling and JNK/c-Jun activation form a pathological feedback loop: D1 receptor stimulation induces JNK phosphorylation,^155^ which can further perturb dopaminergic neurotransmission. Interrupting this feedback loop may provide another therapeutic strategy in PD.

In summary, extensive evidence implicates the JNK-c-Jun axis in multiple pathological processes underlying PD, including oxidative stress, inflammation, and apoptosis. Given c-Jun’s central role as an executioner of stress signals in neurons, this pathway presents a compelling target for therapeutic development. Further elucidation of the molecular details of c-Jun regulation and function in PD will facilitate the identification of new drug targets and the refinement of inhibitors targeting the JNK/c-Jun pathway (Figure 3). Such advances could pave the way for innovative therapies aimed at protecting dopaminergic neurons and slowing the progression of PD. However, long-term pathway inhibition may carry safety liabilities because c-Jun signaling can also participate in adaptive stress responses; therefore, therapeutic development should prioritize CNS-targeted delivery and isoform-selective modulation.

## Conclusion and future perspectives

c-Jun, a component of the AP-1 TF complex, has emerged as a key mediator in the pathogenesis of major neurodegenerative disorders. In AD, PD, and HD, the JNK pathway—particularly the neuron-enriched JNK3 isoform—is aberrantly activated, linking diverse pathogenic stimuli to cellular dysfunction.^156^^,^^157^ Activated JNK phosphorylates c-Jun, driving transcriptional programs that underlie neuronal death, inflammation, and synaptic pathology in these diseases.

A prominent role of c-Jun is in promoting neuronal apoptosis. JNK-mediated phosphorylation of c-Jun triggers the expression of pro-apoptotic genes, and JNK can directly activate intrinsic death effectors (e.g., *BIM*), thereby committing neurons to apoptosis.^156^ Experimental models demonstrate that excessive c-Jun activity is causally linked to neuron loss. For example, in a toxin-based HD model, overexpression of a dominant-negative (non-phosphorylatable) c-Jun in striatal neurons significantly inhibited cell death.^158^ Likewise, JNK3 activation is required for dopaminergic neuron apoptosis in PD models, as pharmacological JNK inhibition blocks toxin-induced dopaminergic cell loss.^156^ Consistently, genetic ablation of JNK3 in an AD mouse model reduced neuronal death and ameliorated cognitive decline.^156^^,^^159^ These findings highlight c-Jun/AP-1 as a pivotal executor of programmed cell death across neurodegenerative diseases.

c-Jun also contributes to chronic neuroinflammation that exacerbates neurodegeneration. Activated AP-1 (c-Jun/c-Fos) can induce expression of inflammatory cytokines and stress-responsive genes in glia. JNK/c-Jun signaling is essential for microglial and astrocytic responses to pathology, forming a feedforward loop between neuron injury and inflammation. Inhibition of JNK has been shown to attenuate the release of pro-inflammatory mediators; notably, a JNK inhibitor, tanzisertib, reduced TNF-α production in an endotoxin-induced neuroinflammatory model.^160^ Conversely, emerging evidence indicates that epitranscriptomic dysregulation of c-Jun’s pathway can heighten inflammation. For instance, loss of the N^6^-methyladenosine (m^6^A) RNA methylation reader, *YTHDF2*, in astrocytes stabilizes *MAP2K4* (an upstream JNK kinase) transcripts, leading to hyperactivation of the JNK-c-Jun cascade and excessive pro-inflammatory signaling.^160^^,^^161^^,^^162^^,^^163^^,^^164^ Such findings illustrate how post-transcriptional mechanisms modulate c-Jun activity in glial inflammation and suggest that restoring these regulatory checkpoints could mitigate neuroinflammatory damage.

In addition, the JNK-c-Jun axis is implicated in synaptic dysfunction, an early and critical feature of neurodegenerative disorders. In AD, JNK is aberrantly activated at synapses in the hippocampus, contributing to the removal of postsynaptic proteins and glutamate receptors, and resulting in synaptic weakening.^41^ c-Jun activation downstream of JNK3 can facilitate core AD pathologies that disrupt synapses: phosphorylation of c-Jun and other substrates by JNK3 promotes tau protein hyperphosphorylation and tangle maturation, and JNK3 phosphorylates APP to enhance Aβ production.^156^ These changes drive synaptic loss and cognitive deficits. Correspondingly, blocking JNK signaling preserves synaptic integrity. In APP transgenic AD mice, a peptide inhibitor of JNK (D-JNKI1) prevented the loss of postsynaptic density components and dendritic spines, rescuing synaptic function.^41^ Similar mechanisms appear operative in HD, where aberrant JNK3 activation impairs axonal transport by phosphorylating motor proteins, contributing to synaptic failure. In a chemical model of HD, inhibition of c-Jun activation protected axonal trafficking and synaptic connectivity.^158^ Thus, c-Jun links pathogenic protein aggregates and stress signaling to synaptic dysfunction in these diseases.

Given c-Jun’s broad influence on neuronal survival and connectivity, the JNK-c-Jun pathway is an attractive therapeutic target for neurodegenerative diseases. In multiple models of AD and PD, JNK inhibition has produced neuroprotective effects. The pan-JNK inhibitor SP600125, for instance, not only attenuated dopaminergic neuron loss in an MPTP mouse model of PD but also reduced amyloid plaque load and neurofibrillary tangle formation in AD models.^160^ Such inhibitors can interrupt the apoptotic and degenerative programs driven by c-Jun. Further supporting this approach, c-Jun activation is observed in degenerating neurons in patients with AD and PD, directly linking this stress pathway to disease pathology. Accordingly, JNK3-selective compounds are being developed to maximize efficacy in the CNS while minimizing off-target effects. One brain-penetrant JNK3 inhibitor was shown to improve learning and memory in AD model mice, coinciding with reduced Aβ plaque burden and tau phosphorylation.^156^ Similarly, peptide inhibitors such as D-JNKI1 have demonstrated synapto-protective and anti-apoptotic benefits *in vivo*.^41^ *In vitro* and *in vivo* studies of compound DN-1289 showed reduced p-c-Jun activity in acute optic nerve crush (ONC) injury and ALS *SODI* models, lowered *NFL* levels, and modulation of ALS biomarkers *in vivo*.^165^ Similarly, compound 14 exhibited high *DLK/LZK* inhibitory activity, effective p-c-Jun modulation in cell assays, and good *in vitro* microsomal stability and MDR1-MDCK permeability.^166^ These therapeutic findings highlight that dampening c-Jun activation, often via JNK inhibition, can spare neurons and synapses from programmed cell death and potentially slow disease progression. Therapeutically, several intervention points within the JNK-c-Jun network are being explored, including ATP-competitive JNK inhibitors, peptide-based inhibitors of JNK signaling, and upstream modulation of DLK/LZK-dependent injury signaling. However, translation remains challenging because c-Jun and JNK signaling also contribute to essential stress-adaptation and homeostatic programs; therefore, therapeutic strategies will likely require isoform selectivity (e.g., JNK3-enriched targeting), CNS penetration, and careful control of dose and treatment timing to minimize off-target effects and long-term toxicity.

Although many factors contribute to neurodegeneration, c-Jun stands out as a convergent downstream mediator and linchpin in these disease pathways. It funnels diverse cellular insults, such as misfolded proteins, oxidative stress, and inflammation, into gene programs that drive neuronal dysfunction and death. This makes c-Jun a prime therapeutic target, with clear promise but important translational constraints: modulating c-Jun signaling could interrupt common neuronal death programs. Encouragingly, research shows that dampening c-Jun activity protects neurons and even rescues impaired neurogenesis. Future strategies may combine kinase inhibitors or gene therapy approaches to fine-tune c-Jun signaling, although achieving this safely and specifically in the human brain remains challenging. Overall, c-Jun serves as a unifying hub of neurodegeneration, and targeting its network could broadly bolster neuronal resilience.

## Data and code availability

All data generated or analyzed during this study are included in this published article.

## Acknowledgments

The author(s) declared that financial support was received for the research and/or publication of this article. This work was supported by the 10.13039/501100001809National Natural Science Foundation of China (no: 82474264, no: 82205044, no: 82174170); Basic Research on the Intervention of Bushen Yiqi Formula in Several Airway Inflammatory Diseases (no: 2022QD056), and the Tianshan Talents training program of Xinjiang Province (2023TSYCJC0048).

## Author contributions

F.A.K.: Conceptualization, formal analysis, investigation, methodology, writing – original draft, writing – review and editing, and construction of figures and tables. H.K., U.A.A., M. Nurahmat, M. Nabijan: Writing – review and editing and construction of figures. J.D., M.A.: Supervision, funding acquisition, project administration, resources, validation, writing – review and editing. All authors listed have made a substantial, direct, and intellectual contribution to the work and have approved it for publication.

## Declaration of interests

The authors declare that the research was conducted in the absence of any commercial or financial relationships that could be construed as a potential conflict of interest.
